# Anti-microbial resistance: the problem is now or in the future

**DOI:** 10.1093/haschl/qxaf027

**Published:** 2025-02-21

**Authors:** Venu Gopal Jonnalagadda

**Affiliations:** Centre for Cellular & Molecular Platforms (C-CAMP), National Centre for Biological Sciences (NCBS), Bugworks Research India Pvt. Ltd., Bangalore 560065, India

**Keywords:** anti-microbial resistance, artificial intelligence, clinical trials

Dear Editor,

I read with great interest the article titled “Sustainable Solutions to the Continuous Threat of Antimicrobial Resistance” by Spellberg et al.^1^ The authors provide a compelling overview of the multifaceted challenges posed by antimicrobial resistance (AMR) and offer actionable strategies to address this growing crisis. As AMR continues to threaten global health security, the need for sustainable, collaborative solutions has never been more urgent. I would like to appreciate the authors for their comprehensive analysis and highlight a few areas that could benefit from further emphasis.

The article rightly underscores the importance of responsible antibiotic use in both human medicine and agriculture. However, I believe greater attention should be given to the role of behavioral change and public awareness campaigns in achieving this goal. While healthcare providers and policymakers play a critical role, the public must also be educated about the dangers of antibiotic misuse, such as self-medication and the demand for antibiotics for viral infections.^2^ Community-based initiatives and digital health tools could be leveraged to disseminate this information effectively.

Additionally, the discussion on investing in research and development (R&D) for new antibiotics and alternative therapies is timely. However, the article could explore the potential of open-access platforms and global partnerships to accelerate innovation. For instance, initiatives like the Global Antibiotic Research and Development Partnership (GARDP) and the Combating Antibiotic-Resistant Bacteria Biopharmaceutical Accelerator (CARB-X) are excellent examples of collaborative efforts that could serve as models for future R&D endeavors. Importantly, CARB-X is funding for preclinical and healthy volunteer studies (Phase I), including therapeutics, vaccines, and diagnostics, while GARDP is supporting later clinical stage developments (Phases II and III).^3^

The article's emphasis on strengthening surveillance systems is commendable, but it could further highlight the role of artificial intelligence (AI) and machine learning in enhancing AMR surveillance. These technologies can analyze mammoth datasets to predict resistance patterns, identify emerging threats, and inform targeted interventions. Integrating AI into national and global surveillance networks could significantly improve our ability to combat AMR.

In conclusion, the article provides a valuable roadmap for tackling AMR, but sustained global commitment and innovation are essential to turn these strategies into tangible outcomes. I urge researchers, policymakers, and stakeholders to prioritize AMR as a global health priority and work collaboratively to ensure a sustainable future for antimicrobial therapies on a *Si Opus Sit* basis.
